# Effectiveness of Flexible Bronchoscopy Simulation-Based Training: A Systematic Review

**DOI:** 10.1016/j.chest.2023.05.012

**Published:** 2023-05-12

**Authors:** Eveline C.F. Gerretsen, Aoben Chen, Jouke T. Annema, Marleen Groenier, Erik H.F.M. van der Heijden, Walther N.K.A. van Mook, Frank W.J.M. Smeenk

**Affiliations:** a Department of Educational Development and Research, School of Health Professions Education (SHE), Maastricht University, Maastricht, The Netherlands; b Department of Respiratory Medicine, Catharina Hospital, Eindhoven, The Netherlands; c Department of Respiratory Medicine, Amsterdam University Medical Centers, Amsterdam, The Netherlands; d Technical Medical Center, University of Twente, Enschede, The Netherlands; e Department of Respiratory Medicine, Radboudumc, Nijmegen, The Netherlands; f Department of Intensive Care, Maastricht University Medical Center+, Maastricht, The Netherlands

**Keywords:** bronchoscopy, learning, simulation, training

## Abstract

**Background:**

The implementation of simulation-based training (SBT) to teach flexible bronchoscopy (FB) skills to novice trainees has increased during the last decade. However, it is unknown whether SBT is effective to teach FB to novices and which instructional features contribute to training effectiveness.

**Research Question:**

How effective is FB SBT and which instructional features contribute to training effectiveness?

**Study Design and Methods:**

We searched Embase, PubMed, Scopus, and Web of Science for articles on FB SBT for novice trainees, considering all available literature until November 10, 2022. We assessed methodological quality of included studies using a modified version of the Medical Education Research Study Quality Instrument, evaluated risk of bias with relevant tools depending on study design, assessed instructional features, and intended to correlate instructional features to outcome measures.

**Results:**

We identified 14 studies from an initial pool of 544 studies. Eleven studies reported positive effects of FB SBT on most of their outcome measures. However, risk of bias was moderate or high in eight studies, and only six studies were of high quality (modified Medical Education Research Study Quality Instrument score ≥ 12.5). Moreover, instructional features and outcome measures varied highly across studies, and only four studies evaluated intervention effects on behavioral outcome measures in the patient setting. All of the simulation training programs in studies with the highest methodological quality and most relevant outcome measures included curriculum integration and a range in task difficulty.

**Interpretation:**

Although most studies reported positive effects of simulation training programs on their outcome measures, definitive conclusions regarding training effectiveness on actual bronchoscopy performance in patients could not be made because of heterogeneity of training features and the sparse evidence of training effectiveness on validated behavioral outcome measures in a patient setting.

**Trial Registration:**

PROSPERO; No.: CRD42021262853; URL: https://www.crd.york.ac.uk/prospero/

FOR EDITORIAL COMMENT, SEE PAGE 820

**Take-home Points**

**Study Question:** How effective is flexible bronchoscopy simulation-based training and which instructional features contribute to training effectiveness?

**Results:** This systematic review shows that flexible bronchoscopy simulation-based training is effective in improving skills when evaluated in a simulation setting. However, the effects of simulation training on skill performance of novices in a patient setting are less clear because of a lack of studies using homogeneous validated outcome measures. Integrating bronchoscopy simulation training programs in the curriculum and increasing task difficulty appear to contribute to training effectiveness.

**Interpretation:** To further improve our knowledge of the effectiveness of bronchoscopy simulation-based training and how to optimize these training programs, we advocate that future studies use more homogeneous validated outcome measures, preferably in a patient setting.

Use of simulation in health professions education has increased significantly over the past 2 decades.^1^ This shift from the traditional apprenticeship model (see one, do one, teach one) toward simulation-based training (SBT) is largely the result of concerns for patient safety.^2^^,^^3^ In general, the apprenticeship method, and more specifically, flexible bronchoscopy (FB) training, are associated with a higher complication risk^4^^,^^5^ and increased patient discomfort.^6^ Hence, a shift to SBT might be desirable.

Currently, a variety of FB simulators are used for bronchoscopy training (eg, animal models,^7^ 3-D printed airway models,^8^ high-fidelity virtual reality simulators^9^^,^^10^). To date, four reviews on bronchoscopy training programs (TPs) using simulators have been published.^2^^,^^11^, ^12^, ^13^ The systematic review by Kennedy et al^2^ concluded that SBT was effective in comparison with no training. The authors also assessed the presence of 10 key instructional features, as identified in an earlier review on features of medical simulation TPs.^14^ The interpretation of the Kennedy et al^2^ review is somewhat hampered by the inclusion of a variety of different simulation methods for different types of bronchoscopies (eg, rigid bronchoscopy, FB, endobronchial ultrasound). Furthermore, the studies’ settings were heterogeneous (eg, in an otolaryngology or anesthesiology setting). Bronchoscopy in these settings requires less detailed navigation competencies compared with FB in a pulmonology setting.^9^ Three additional reviews have been published since then on FB SBT,^11^, ^12^, ^13^ but their interpretation is also hampered by their narrative designs and lack of systematic study quality assessments. In addition, none of these three reviews looked at the effectiveness of instructional features present in the included TPs.

Based on these reviews, there is still no clear-cut answer to the basic question of whether FB SBT is effective in improving basic FB skills of novice pulmonology trainees and which instructional TP features might contribute to training effectiveness. In this review, we therefore aim (1) to summarize the current evidence of the effectiveness of SBT on improving novice bronchoscopists’ basic FB skills, taking into account quality of included studies, and (2) to give an overview of the general and instructional features of the investigated TPs. Furthermore, we describe the relation between instructional features and outcomes to identify the most effective training strategies.

## Study Design and Methods

This review was written in compliance with the Preferred Reporting Items for Systematic Reviews and Meta-Analyses guidelines.^15^ Because only publicly available data were used and no human subjects were involved, institutional review board approval was not required.

A search was performed in PubMed, Embase, Scopus, and Web of Science, encompassing all available articles up until November 10, 2022, using the search strategies developed in collaboration with an experienced research librarian (e-Table 1). The search was composed of relevant terms related to bronchoscopy, simulation training, and competence. No language criteria were applied. The following selection criteria were used for inclusion of studies into the final analysis: (1) the study design had to be a pretest-posttest, two-group nonrandomized, or randomized design; (2) the study had to include novice trainees regarding bronchoscopy experience; and (3) the intervention had to include at least basic FB SBT, where the simulator is a tool or device with which the trainee physically interacts to simulate an FB. Studies reporting only trainee-reported outcome measures were excluded.

Two reviewers (E. C. F. G. and A. C.) independently performed all evaluations regarding screening and data extraction. Only full texts were considered. In case of discrepancy, a consensus meeting was planned. In case no consensus could be achieved, a third reviewer (F. W. J. M. S.) made the final decision.

First, the reviewers screened all titles and abstracts of studies from the search results against the inclusion criteria. After achieving consensus on which articles to include, they screened reference lists of those articles for other possible relevant articles.

Second, the following characteristics of the full texts of included papers were assessed: study design, number of participants and their level of education, simulator modality, comparator, outcome measures, and intervention’s effects on the outcome measures.

Articles that fully met all inclusion criteria were included for analysis.

The reviewers also evaluated on which Kirkpatrick level^16^ outcome measures were assessed. This is a four-level model to evaluate training impact: reaction (level 1), learning (level 2), behavior (level 3), and results (level 4).^17^ In a simulation training setting, level 1 refers to participants’ satisfaction with the training (not applicable in our study because these studies were excluded), level 2 refers to an improvement in skills (an improvement in outcomes in a simulation setting), level 3 learning is suggested when on-the-job behavior is improved (an improvement in bronchoscopy performance in a patient setting), and level 4 refers to improvement in patient outcomes^18^ (eg, less discomfort, fewer complications).

To prevent bias, the name of the journal, authors, abstract, and discussion sections were removed from the articles for the three reviewers in all their further evaluations. The reviewers then assessed the methodological quality of studies using the modified Medical Education Research Study Quality Instrument (mMERSQI).^19^ A score of 4.5 to 8.5 indicates low quality, 9.0 to 13.0 indicates moderate quality, and 13.5 to 18.0 indicates high quality.^20^ This tool was adapted on the validity of the evaluation instrument domain because this domain was considered not fully applicable for the current review because of it being open to interpretation in this setting. Therefore, this domain was transformed into a single known-groups comparison parameter to evaluate the validity of the evaluation instrument, for which a positive score was given if the instrument had any (referred) proven validity in terms of a known-groups comparison. Considering the maximum score with our mMERSQI tool was 2.0 points lower than the original one, we adapted the interpretation of the scores regarding quality accordingly: 4.5 to 8.0 indicating low quality, 8.5 to 12.0 indicating moderate quality, and 12.5 to 16.0 indicating high quality.

Risk of bias (RoB) was determined with different tools depending on study design^21^^,^^22^ (Table 1). For each study, the reviewers calculated how many items they could answer positively, where a positive score for an item means that the study had a low RoB for that item. Next, they divided the total number of positive items by the number of applicable items for that study and transformed all scores to a final score on the original scale of the RoB tool.

**Table 1:** Risk of Bias Tool Used for Each Study Design

Study Design	Risk of Bias Tool	Study	Maximum Score
Pretest-posttest	Quality Assessment Tool for Before-After (Pre-Post) Studies With No Control Group	National Heart, Lung, and Blood Institute^22^	12
Two-group nonrandomized	Critical Appraisal Tool for Quasi-Experimental Studies (nonrandomized experimental studies)	Tufanaru et al^21^	9
Randomized controlled trial	Quality Assessment of Controlled Intervention Studies	National Heart, Lung, and Blood Institute^22^	14

Finally, all studies were carefully assessed for the general and instructional features listed in Table 2. Features not explicitly mentioned in a study were assumed not to be present. In case the reviewers could not extract all characteristics from the publication, they contacted the authors to request further information.

**Table 2:** General and Instructional Features and Definitions

Feature Category	Feature	Definition
General	Duration	Training duration in hours and days
	Assessment by	Assessment by simulator, observer, or both
	Observer instruction	Observer instruction described
	Validity evidence reported/referred	Use of validated assessment tool/procedure or referred to known-groups comparison for the assessment tool/procedure
Instructional design^a^	Clinical variation	Multiple different scenarios were present
	Curriculum integration	Training was a part of the curriculum
	Feedback	Feedback was provided by an instructor
	Group practice	Training occurred in a group
	Individualized learning	Training could be tailored to the trainee depending on individual performance
	Mastery learning	Trainee must attain a predefined level of performance
	Prestudy	Participants had to study or watch a video or presentation before the training
	Range in task difficulty	There was a variation in task difficulty

a These features were partially based on a study from Issenberg et al^14^ from 2005. Although initially planned, it was decided to leave out the following three features: (1) multiple learning strategies (because no clear-cut definition of a learning strategy could be found), (2) number of learning modalities (because if training programs included more learning modalities, it was always because of videos or books being present, which was already taken into account in prestudy), and (3) repetition (because the opportunity to repeat a task multiple times is almost always possible when training on a simulator).

Although a meta-analysis was planned, this proved impossible because of the high level of heterogeneity of the interventions and outcomes in the included studies.^23^ Therefore, the reviewers evaluated the methodological quality and characteristics of all studies and related these to their results.

## Results

**Figure 1:**
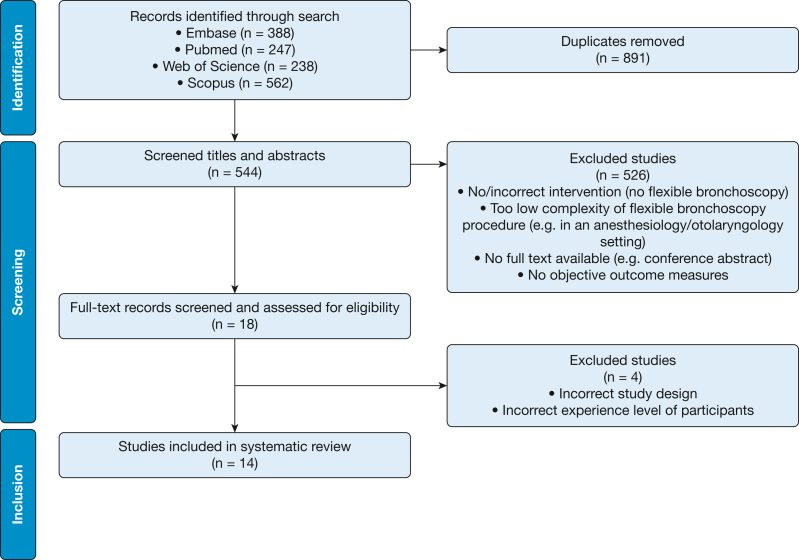
Flow diagram of the systematic review.

The search yielded 544 articles after removal of duplicates. Initially, 18 studies ended up meeting the inclusion criteria (Fig 1). Reference list analysis of those studies did not lead to any other relevant articles. After evaluating the full texts of these studies, the reviewers were undecided about five studies. The third reviewer excluded four of those studies because the design or the participants’ experience level did not meet the inclusion criteria.^24^, ^25^, ^26^, ^27^

Methodological quality of studies was moderate to high, with mMERSQI scores ranging from 10 to 14 on a 16-point scale (mean ± SD, 12.2 ± 1.2) (Table 3).^6^^,^^9^^,^^10^^,^^28^, ^29^, ^30^, ^31^, ^32^, ^33^, ^34^, ^35^, ^36^, ^37^, ^38^ Six studies had a high mMERSQI score (≥ 12.5).^28^, ^29^, ^30^, ^31^, ^32^, ^33^ The score differences were mainly caused by differences in study design.

**Table 3:** Study Characteristics of Included Studies

Study	Design	No. of Participants (IG/CG)^a^	Experience Level	Simulator Modality	Comparator	Outcome Measures	mMERSQI
Colt et al^6^	P-P	5	Novice pulmonary and critical care medicine fellows	VR	NA	Simulator (learning)	11
Ost et al^28^	RCT	6 (3/3)	Novice pulmonary fellows	VR	Conventional training	Patient (behavior)	13
Blum et al^29^	RCT	10 (5/5)	First-year surgical residents	VR	No training	Patient (behavior)	13
Wahidi et al^33^	2G-NR	44 (22/22)	Novice pulmonary fellows	VR	Conventional training	Patient (behavior)	14
Colt et al^31^	P-P	24	Novice pulmonary and critical care fellows	Unknown	NA	Simulator (learning)	13
Bjerrum et al^35^	P-P	47	Medical students	VR	NA	Simulator (learning)	12
Krogh et al^32^	RCT	20 (10/10)	Medical students	VR	No training	Simulator (learning)	13.5
Bjerrum et al^10^	P-P	36	Medical students	VR	NA	Simulator (learning)	12
Bjerrum et al^36^	P-P	20	Physicians in training	VR	NA	Simulator (learning)	12
Gopal et al^37^	P-P	47	Medical students	VR	NA	Simulator (learning)	11.5
Veaudor et al^9^	P-P	8	Novice first-year pulmonology residents	VR	NA	Simulator (learning)	10
Feng et al^34^	P-P	28	Medical students	Part-task trainer	NA	Simulator (learning)	11
Schertel et al^38^	P-P	54	Medical students	VR	NA	Simulator (learning)	11
Siow et al^30^	2G-NR	18 (8/10)	Pulmonary medicine residents	VR	Conventional training	Patient (behavior)	14

2G-NR = two-group nonrandomized; CG = control group; IG = intervention group; mMERSQI = modified Medical Education Research Study Quality Instrument; NA = not applicable; P-P = pretest-posttest; RCT = randomized controlled trial; VR = virtual reality.

a For pretest-posttest studies, only one number is shown because those studies do not have a CG.

Table 3 shows study characteristics. Most (n = 9) used a pretest-posttest design, and the number of participants in all included studies ranged from five to 54. Twelve studies used a virtual-reality simulator,^6^, ^9^, ^10^, ^28^, ^29^, ^30^, ^32^, ^33^, ^35^, ^36^, ^37^, ^38^ one study used a part-task trainer,^34^ and for one study, the reviewers could not extract the used simulation equipment from the text.^31^ Ten studies measured outcomes in a simulation setting^6^^,^^9^^,^^10^^,^^31^^,^^32^^,^^34^, ^35^, ^36^, ^37^, ^38^ (eg, number of wall contacts, [modified] validated Bronchoscopy Skills and Tasks Assessment Tool [BSTAT]).^39^ Four studies measured Kirkpatrick (behavioral) level 3 outcomes (eg, BSTAT for a bronchoscopy performed on a patient).^28^, ^29^, ^30^^,^^33^

RoB scores of included studies are described in Table 4. RoB scores of pretest-posttest studies ranged from 4.4^37^^,^^38^ to 9.6^36^ on a 12-point scale (mean ± SD, 6.4 ± 1.8). Only two studies^10^^,^^36^ had relatively high RoB scores (8.4 and 9.6) and were therefore considered to have a low RoB. The two two-group nonrandomized design studies^30^^,^^33^ had a low RoB (final score of 7 on a 9-point scale). The three randomized controlled trials had a moderate to low RoB, with scores ranging from 7.0^28^ to 10^32^ on a 14-point scale.

**Table 4:** Overview of Studies’ Risk of Bias Scores

Design	Study	Positive Items	Applicable Items	Final Score
Pretest-posttest	Colt et al^6^	5	11	5.5
(maximum score 12)	Colt et al^31^	6	11	6.5
	Bjerrum et al^35^	6	10	7.2
	Bjerrum et al^10^	7	10	8.4
	Bjerrum et al^36^	8	10	9.6
	Gopal et al^37^	4	11	4.4
	Veaudor et al^9^	5	11	5.5
	Feng et al^34^	6	11	6.5
	Schertel et al^38^	4	11	4.4
Two-group nonrandomized	Wahidi et al^33^	7	9	7.0
(maximum score 9)	Siow et al^30^	7	9	7.0
Randomized controlled trial	Ost et al^28^	7	14	7.0
(maximum score 14)	Blum et al^29^	9	14	9.0
	Krogh et al^32^	10	14	10.0

The final score was calculated by dividing the number of positive items by the number of applicable items, transformed to the original maximum possible score of the risk of bias tool. Pretest-posttest study scores were transformed to a final score on a 12-point scale, two-group nonrandomized study scores were transformed to a final score on a 9-point scale, and randomized controlled trial design study scores were transformed to a final score on a 14-point scale.

Table 5 shows general features of included studies. There was a large variation in the duration of TPs, ranging from 45 min^34^ in 1 day to 12 h in 12 weeks.^30^ Five TPs lasted > 1 day.^6^^,^^28^^,^^30^^,^^36^^,^^37^ Trainees were assessed only on the simulator in four studies.^10^^,^^35^^,^^36^^,^^38^ Of the studies where an observer was (partially) included in the assessment methods, four described whether the observer was instructed on how to assess the trainees.^9^^,^^31^, ^32^, ^33^ Studies that included assessment tools used a validated version of the BSTAT,^33^^,^^34^ a modified version of the BSTAT,^30^^,^^31^^,^^37^ or another validated bronchoscopy assessment tool.^32^

**Table 5:** Overview of General Features of Included Studies

Study	Duration	Assessment by	Observer Instruction	Validity Evidence Reported/Referred
Colt et al^6^	> 1 d	Both	Unknown	No
Ost et al^28^	> 1 d	Observer	Unknown	No
Blum et al^29^	< 1 h and > 1 h, 1 d	Observer	Unknown	No
Wahidi et al^33^	Unknown	Observer	Yes	Yes
Colt et al^31^	> 1 h, 1 d	Observer	Yes	No
Bjerrum et al^35^	> 1 h, 1 d	Simulator	NA	Yes
Krogh et al^32^	< 1 h and > 1 h, 1 d	Observer	Yes	Yes
Bjerrum et al^10^	> 1 h, 1 d	Simulator	NA	Yes
Bjerrum et al^36^	> 1 h, 1 d, and > 1 d	Simulator	NA	Yes
Gopal et al^37^	> 1 d	Observer	Unknown	No
Veaudor et al^9^	Unknown	Both	Yes	Yes
Feng et al^34^	< 1 h, 1 d	Observer	Unknown	Yes
Schertel et al^38^	< 1 h, 1 d	Simulator	NA	No
Siow et al^30^	> 1 d	Observer	Unknown	No

NA = not applicable.

Instructional features of included studies are described in Table 6. Apart from clinical variation (present in nine studies) and prestudy (present in 10 studies), there was no dominant pattern of any of the other instructional features.

**Table 6:** Overview of Instructional Features of Included Studies

Study	Clinical Variation	Curriculum Integration	Instructor Feedback	Group Practice	Individualized Learning	Mastery Learning	Prestudy	Range in Task Difficulty
Colt et al^6^	Yes	No	No	No	No	No	Yes	No
Ost et al^28^	Yes	No	No	No	No	No	Yes	No
Blum et al^29^	Yes	No	No	No	No	No	No	No
Wahidi et al^33^	No	Yes	No	No	No	No	No	Yes
Colt et al^31^	Yes	No	Yes	Yes	No	No	Yes	Yes
Bjerrum et al^35^	Yes	No	Yes	No	No	No	Yes	No
Krogh et al^32^	Yes	No	No	No	No	No	Yes	Yes
Bjerrum et al^10^	Yes	No	Yes	Yes	No	No	Yes	No
Bjerrum et al^36^	Yes	No	No	No	No	No	Yes	No
Gopal et al^37^	No	No	No	No	No	No	No	No
Veaudor et al^9^	No	No	No	No	No	No	Yes	No
Feng et al^34^	No	No	No	No	No	No	Yes	No
Schertel et al^38^	No	No	Yes	No	No	No	Yes	Yes
Siow et al^30^	Yes	Yes	No	No	No	No	No	Yes

Table 7 shows outcome measures that were present in two or more studies. We only reported these outcome measures for clarity, given the abundance of other outcome measures that were only present once in included studies (a complete overview of all outcome measures can be found in e-Table 2). Eleven studies reported significant improvements in more than one-half of their outcome measures. Outcome measures were heterogeneous, ranging from simulator metrics (eg, percentage of time in midlumen) to (validated) bronchoscopy assessment tool end scores. Two of four studies with outcomes on Kirkpatrick level 3 reported significant improvements in (modified) BSTAT outcomes.^30^^,^^33^ Ost et al,^28^ Blum et al,^29^ and Siow et al^30^ all reported procedure time outcomes in a patient setting. However, their effect on procedure time was conflicting.

**Table 7:** Overview of Outcome Measures

Outcome Measure	Level	Studies
Procedure time	2	Colt et al^6^^,^^b^; **Bjerrum et al****^35^**; **Krogh et al****^32^**^**,**^^a^; **Bjerrum et al****^10^**; **Bjerrum et al****^36^**; **Veaudor et al****^9^**
Segments entered	2	Bjerrum et al^35^; **Bjerrum et al****^10^**; **Bjerrum et al****^36^**; **Feng et al****^34^**^**,**^^a^
Time in redout	2	Colt et al^6^; Bjerrum et al^35^; **Bjerrum et al****^10^**; **Bjerrum et al****^36^**
Wall contacts	2	**Bjerrum et al****^35^**; **Bjerrum et al****^10^**; Bjerrum et al^36^
(M)BSTAT simulator	2	**Colt et al****^31^**^**,**^^a^; **Gopal et al****^37^**^**,**^^a^; **Feng et al****^34^**^**,**^^a^
% segments entered	2	Bjerrum et al^35^; **Bjerrum et al****^10^**; **Bjerrum et al****^36^**
% segments entered/min	2	**Bjerrum et al****^35^**; **Bjerrum et al****^10^**; **Bjerrum et al****^36^**
Segments correctly identified	2	**Veaudor et al****^9^**^**,**^^a^; **Schertel et al****^38^**
Segments correctly visualized and identified/procedure time	2	**Ost et al****^28^**^**,**^^a^; **Veaudor et al****^9^**^**,**^^a^
Segments missed	2	**Colt et al****^6^**^**,**^^b^; **Schertel et al****^38^**
% time midlumen	2	Veaudor et al^9^; Schertel et al^38^
% time scope-wall contacts	2	Veaudor et al^9^; Schertel et al^38^
Procedure time	3	**Ost et al****^28^**^**,**^^a^; Blum et al^29^^,^^a^; Siow et al^30^^,^^a^
(M)BSTAT patient	3	**Wahidi et al****^33^**^**,**^^a^; **Siow et al****^30^**^**,**^^a^

Studies indicated in boldface font showed a significant improvement in the listed outcome measure. (M)BSTAT = Modified Bronchoscopy Skills and Tasks Assessment Tool.

a Outcome recorded via direct observation (ie, an observer instead of simulator metrics).

b Outcome both recorded via direct observation and via simulator metrics.

When evaluating the study characteristics of the studies with the highest quality (mMERSQI > 12) and positive results on the most relevant outcome measures (higher than Kirkpatrick level 2), we found that these studies^30^^,^^33^ shared the following characteristics: a gradual increase in task difficulty and integration of the TP in the curriculum.

## Discussion

This review showed that FB SBT is an effective training method to teach basic bronchoscopy skills to novice trainees. The study quality of most studies was moderate to high. Despite these positive results, evidence for positive effects on Kirkpatrick levels 3 and 4 is still scarce. Finally, including a range in task difficulty and integrating the TP in the curriculum seem to be important to teach novices bronchoscopy skills that lead to improved bronchoscopy performance in a patient setting.

### Study Design

Studying the effects of FB SBT is complex: because of the nature of the intervention and for ethical reasons, designing a blinded randomized controlled trial is difficult. Therefore, most included studies used a pretest-posttest design. This design has some drawbacks, the main being a pretest effect,^40^ meaning that performing a pretest might influence the scores a trainee obtains on the posttest. This testing effect might have led to an overestimation of those studies’ reported results. None of the studies in this review corrected for this possible pretest effect.

A review on postgraduate medical education simulation boot camps for clinical skills also reported that most studies used a single group pretest-posttest design, limiting the strength of the effectiveness of the reported interventions.^41^ This was also the case in a systematic review on technology-enhanced simulation for health professions education, where most studies used a pretest-posttest design.^42^ Despite its drawbacks, the pretest-posttest design may be inevitable for investigating FB SBT effectiveness, given the ethical objections associated with some trainees not practicing their skills on a simulator when one is available. However, once this design is chosen, it is important that researchers investigate the extent of a testing effect and adjust for it. In addition, to prevent bias, assessments in these studies should ideally be performed by a blinded observer.

Although long-term retention of FB skills is crucial, only one study measured participants’ skills retention after training over a period of > 6 months.^33^ This lack of studies measuring skill retention over a longer period of time after simulation training was also noticed in surgery and emergency care.^43^^,^^44^ However, in a previous review on critical care SBT, several studies were found evaluating retention outcomes using validated assessment methods after simulation training.^45^ Another study on SBT for internal medicine residents even reported both simulation retention outcomes and retention outcomes measured in a patient setting.^46^ Preferably, future studies on FB SBT should measure trainees’ skill acquisition longitudinally, where possible in a patient setting.

### Outcome Measures

Ideally, SBT leads to positive outcomes on Kirkpatrick level 4 (eg, therapeutic/diagnostic completeness, complications, patient comfort); however, no studies in the current review reported outcomes at this level. It is difficult to design a study investigating the effect of SBT on patient outcomes from both an ethical and practical point of view, and potentially irregular links between simulation interventions and patient outcomes may exist.^47^^,^^48^

There was no consensus among investigators on outcome measures: a wide variety was used, with some simulator-generated and others observer-related. Moreover, although five studies used a (modified) BSTAT, only two studies used a validated version.^33^^,^^34^ In addition, all studies used a different version, leading to considerable heterogeneity, even among these studies. This problem was also identified in reviews of other areas of medical simulation training research (eg, training for surgical skills, ophthalmology, laparoscopy, endoscopy), where included studies varied highly in outcomes and assessment methods.^49^, ^50^, ^51^ To overcome this problem of heterogeneity and enable comparisons between studies, it is of great importance that future studies use validated homogeneous outcome measures, most preferably at a patient level (Kirkpatrick level 3 or 4). Patients having to undergo a bronchoscopy will be most interested in an adequately performed and complete bronchoscopy with the highest diagnostic and/or therapeutic yield, in preferably the shortest duration possible. Therefore, assessing trainees with a previously validated qualitative assessment (eg, validated version of the BSTAT) combined with procedure time as a secondary outcome measure will probably be very relevant to evaluate basic bronchoscopy skills. Structured progress, being the number of times an operator progressed from one segment to the correct next segment during bronchoscopy, might be added as well, because one study reported strong validity evidence of its use.^52^

### Instructional Features

Curriculum integration and a range in task difficulty seemed to be relevant when evaluating the two studies with the highest quality.^30^^,^^33^ Several bronchoscopy TPs have already incorporated SBT in their curriculum,^53^^,^^54^ and some fellowships in interventional pulmonology require SBT.^55^ Unfortunately, no studies to date showed that curriculum integration had a positive effect on residents’ functioning at a behavioral level (Kirkpatrick level 3). Together with only two studies in this review that implemented their TP in the curriculum, it seems that no well-founded conclusions about the importance of curriculum integration can be drawn. However, we regard not integrating simulation training in the curriculum as ethically questionable. Unlike the apprenticeship method, SBT allows trainees to climb the initial, steep part of the learning curve of improving their bronchoscopy skills outside the patient setting. This results in lower stress levels for the trainee and, more importantly, less patient discomfort and morbidity compared with the apprenticeship method,^11^^,^^37^^,^^56^ which makes mandatory SBT for all trainees ethically desirable. Laparoscopic and cardiac bedside skill TPs have implemented simulations of a range in difficulty,^57^ and their relevance is also in line with an earlier review investigating the effectiveness of instructional design features in SBT,^58^ where a positive pooled effect of simulations with a range of difficulties was reported on behavior and patient outcomes.^58^ This is in line with previous research, which showed that competence cannot be indicated solely by a high number of performed procedures^59^ and where escalating task difficulty might be important to gaining competence. Nevertheless, only five studies in this review used a range of task difficulties in their program, making evidence of its relevance in an FB SBT setting rather sparse.

According to previous research, most bronchoscopy learners prefer to directly apply their newly acquired knowledge and skills^60^ in practice. Therefore, simulation TPs should preferably be integrated in an experiential learning model, with case-based learning exercises and small groups with a low trainee-to-instructor ratio enabling frequent interaction and feedback.^60^ However, given the sparse evidence on the actual effectiveness of these instructional features in a bronchoscopy training setting, more research into their relevance for FB SBT programs is warranted.

### Strengths and Limitations

This review has several strengths. It provided a comprehensive overview of current evidence on FB SBT effectiveness in improving FB skills for novice bronchoscopists. It focused solely on FB, and in contrast with previous recent research, study quality, RoB, and present instructional features were evaluated. Articles in any language were considered, and multiple databases were used for the literature search. Reviewers were blinded when they assessed study quality, general features, instructional features, and outcomes, and all assessments were performed independently.

This review also has several limitations. First, because of heterogeneity in the simulation interventions and outcome measures, no formal meta-analysis could be performed. This made it impossible to compare study outcomes quantitatively and to calculate pooled effect sizes of instructional features. Second, the number of included studies was relatively small, which limited the ability to formulate well-founded, qualitative conclusions about the relevance of instructional features. Third, studies measuring outcomes only on Kirkpatrick level 1 were excluded. Although satisfaction with the training can be important for building participants’ self-confidence, this outcome measure was considered less relevant for the purpose of this review. Furthermore, we found only one Kirkpatrick level 1 study that met the inclusion criteria.^61^ Fourth, the methods developed by the National Heart, Lung, and Blood Institute^22^ and Tufanaru et al^21^ used to calculate RoB of studies are not yet validated. Finally, it was decided to adapt the MERSQI for the purposes of this review because some parameters were found to be open to interpretation in this setting. Although this adjustment can raise questions about the validity of the MERSQI for this use, we suspect the possibility of bias to be small because these items involve at maximum only three of the 18 points that can be scored on the MERSQI.

## Interpretation

SBT is effective in teaching novices basic bronchoscopy skills. Including a gradual increase in task difficulty seems to be important when designing a TP and integrating the TP into the curriculum. However, evidence for effectiveness on a behavioral (Kirkpatrick level 3) and patient level (Kirkpatrick level 4) is scarce. Future studies should therefore focus on using validated homogeneous outcome measures focused on these levels.

## Funding/Support

Funded by the Catharina Hospital research fund and the board of directors of Maastricht University Medical Center+.

## Financial/Nonfinancial Disclosures

None declared.

## Supplementary Data

e-Online Data

Audio
