# Supplementary material for: Effectiveness of Flexible Bronchoscopy Simulation-Based Training: A Systematic Review
Source: Chest. 2023 May 12;164(4):952–62. doi: 10.1016/j.chest.2023.05.012 (PMC10645598; doi:10.1016/j.chest.2023.05.012)
Supplement: e-Online Data [file mmc1.docx]

**Supplemental material**

e-Table 1:

The full search strategies for studies on the effectiveness of bronchoscopy simulation training.

| Database | Search |
| --- | --- |
| Embase | (exp bronchoscopy/ OR tracheobronchoscop*.ti,ab,kw. OR bronchial endoscop*.ti,ab,kw. OR laryngotracheobronchoscop*.ti,ab,kw. OR bronchoscop*.ti,ab,kw. OR ((bronchi.ti,ab,kw. OR bronchus.ti,ab,kw.) AND imaging.ti,ab,kw.)) AND (exp simulation training/ OR exp computer simulation/ OR computer interface*.ti,ab,kw. OR computer user interface*.ti,ab,kw. OR user computer interface*.ti,ab,kw. OR user-computer interface*.ti,ab,kw. OR simulation-training.ti,ab,kw. OR simulation-based training OR simulation-based learning OR simulation-based education OR virtual realit*.ti,ab,kw. OR ((simulat*.ti,ab,kw. or interactiv*.ti,ab,kw. or self.ti,ab,kw.) and (computer*.ti,ab,kw. or train*.ti,ab,kw. or lear*.ti,ab,kw.))) AND (competence*.ti,ab,kw. OR skill*.ti,ab,kw. OR effectiv*.ti,ab,kw. OR improve*.ti,ab,kw.) |
| Pubmed | (("Bronchoscopy"[Mesh] OR "Bronchi/diagnostic imaging"[Mesh] OR bronchoscop*[tiab]) AND ("Simulation Training"[Mesh] OR "Computer Simulation"[Mesh] OR "User-Computer Interface"[Mesh] OR virtual realit*[tiab] OR ((simulat*[tiab] OR interactiv*[tiab] OR self[tiab]) AND (computer*[tiab] OR train*[tiab] OR lear*[tiab])))) AND ("Clinical Competence"[Mesh] OR competence*[tiab] OR skill[tiab] OR effectiv*[tiab] OR improve*[tiab]) |
| Web of Science | TS=((bronchoscop* OR tracheobronchoscop* OR “bronchial endoscop*” OR laryngotracheobronchoscop* OR ((bronchi* OR bronchus*) AND imag*)) AND (“simulation training” OR “computer simulation*” OR “computer interface*” OR “computer user interface*” OR “user computer interface*” OR “user-computer interface*” OR simulation-training OR “simulation-based training” OR “simulation-based learning” OR “simulation-based education” OR “virtual realit*” OR ((simulat* OR interactive* OR self*) AND (computer* or train* or lear*))) AND (competence* or skill* or effectiv* or improve*)) |
| Scopus | TITLE-ABS-KEY((bronchoscop* OR tracheobronchoscop* OR "bronchial endoscop*" OR laryngotracheobronchoscop* OR ((bronchi* OR bronchus*) AND imag*)) AND ("simulation training" OR "computer simulation*" OR "computer interface*" OR "computer user interface*" OR "user computer interface*" OR "user-computer interface*" OR simulation-training OR "simulation-based training" OR "simulation-based learning" OR "simulation-based education" OR "virtual realit*" OR ((simulat* OR interactive* OR self*) AND (computer* or train* or lear*))) AND (competence* or skill* or effectiv* or improve*)) |

e-Table 2:
Full overview of outcome measures of included studies

| Study | Outcomes |
| --- | --- |
| Colt 2001 | VR simulator   - Procedure time - **Number of wall contacts/minute bronchoscopy** - % time in red-out - **Segments missed**   Inanimate model   - Procedure time - **Segments missed** |
| Ost 2001 | Patient bronchoscopy   - **Procedure time** - Number of segments entered - Number of segments correctly identified - **% of segments visualized and correctly identified/time in seconds** - **Qualitative bronchoscopy nurse score** - Lidocaine used, ml - Coughing episodes - **Meperidine used, mg** |
| Blum 2004 | Patient bronchoscopy   - Procedure time - **Number of verbal assists** - **Number of physical assists** - Incidence of redundant lobar exams - **Thoroughness of exam** - Confidence - Proficiency |
| Wahidi 2010* | Patient bronchoscopy  BSTAT at **5^th^**, **10^th^**, **15^th^**, **20^th^**, **30^th^**, **50^th^**, 75^th^ and **100^th^** bronchoscopy |
| Colt 2011* | Low-fidelity airway model   - **Cognitive skill test** - **mBSTAT** |
| Bjerrum 2013* | Virtual reality simulator   - **% of segments entered/minute** - Red-out in seconds - **Number of wall collisions** - **Procedure time** - % of segments entered |
| Krogh 2013* | Virtual reality simulator   - **Bronchoscopy quality score** - **Procedure time** - Checklist-score |
| Bjerrum 2014* | Virtual reality simulator   - **% of segments entered/minute** - **Red-out in seconds** - **Number of wall collisions** - **Procedure time** - **% of segments entered** |
| Bjerrum 2016* | Virtual reality simulator   - **% of segments entered/minute** - **Red-out in seconds** - Number of wall collisions - **Procedure time** - **% of segments entered** |
| Gopal 2018* | Virtual reality simulator   - **mBSTAT (anatomy part)** - **mBSTAT (bronchoscopy skills part)** |
| Veaudor 2018* | Virtual reality simulator   - **Visualized anatomical structures** - **Correctly identified anatomical structures** - **Procedure time** - **% of segments correctly visualized and identified/time in seconds** - % of time with scope-wall contact - % of time with scope at mid-lumen |
| Feng 2020* | Low-fidelity airway model   - **mBSTAT** - **Proportion of students able to successfully navigate to and enter target segment** |
| Schertel 2021* | Virtual reality simulator   - **Segments correctly identified on 1^st^ attempt** - **Segments correctly identified on any attempt** - **Segments skipped** - % of time in mid-lumen - % of time with scope wall-contact |
| Siow 2021* | Patient bronchoscopy   - **BSTAT score 6 weeks** - **BSTAT score 21 weeks** - **Airway anesthesia score 6 weeks** - **Airway anesthesia score 12 weeks** - Procedure time - Vocal cord anesthesia time - Intubation attempts |

Outcome measures (self-reported outcome measures not included) indicated in bold were significantly improved after the simulation intervention.

* = the majority of the reported outcome measures in the study were significantly improved after the intervention.
